# Differential localization of histone variant TH2B during the first round compared with subsequent rounds of spermatogenesis

**DOI:** 10.1002/dvdy.33

**Published:** 2019-04-19

**Authors:** My‐Thanh Beedle, Traci Topping, Cathryn Hogarth, Michael Griswold

**Affiliations:** ^1^ School of Molecular Biosciences and Center for Reproductive Biology Washington State University Pullman Washington

**Keywords:** chromatin, germ cells, Hist1h2aa, Hist1h2ba, ID4‐eGFP, neonatal, spermatocyte, spermatogenesis, spermatogonia, testis, TH2A, TH2B, WIN 18446, ZBTB16

## Abstract

**Background:**

Male germ cells are unique because they express a substantial number of variants of the general DNA binding proteins, known as histones, yet the biological significance of these variants is still unknown. In the present study, we aimed to address the expression pattern of the testis‐specific histone H2B variant (TH2B) and the testis‐specific histone H2A variant (TH2A) within the neonatal mouse testis.

**Results:**

We demonstrate that TH2B and TH2A are present in a testis‐enriched for undifferentiated spermatogonia. Co‐localization studies with an undifferentiated marker, ZBTB16, revealed that TH2B and ZBTB16 co‐localize in the neonatal testis. Upon the appearance of the primary spermatocytes, TH2B no longer co‐localized with the ZBTB16 positive spermatogonia but were instead detected within the differentiating spermatogonia. This pattern of expression where TH2B and ZBTB16 no longer co‐localize was maintained in the adult testis.

**Conclusion:**

These findings are in contrast to previous studies, which demonstrated that TH2B and TH2A were found only in adult spermatocytes. Our data are in support of a switch in the expression of these variants following the first round of spermatogonial differentiation. These studies reinforce current understandings that spermatogonia within the neonatal mouse testis are inherently different from those residing within the adult testis.

## Key Findings

- Contrary to previous beliefs, testis specific histone variants TH2B and TH2A are also expressed expressed in undifferentiated spermatogonia in the neonatal mouse testis. Upon the appearance of the primary spermatocytes, TH2B switches its expression from spermatogonia to the spermatocyte population. This study reinforces the idea that spermatogonia in the neonatal mouse testis is inherently different than those residing within the adult.

## 1 INTRODUCTION

Mammalian spermatogenesis is a complex process of germ cell differentiation resulting in the production of haploid male gametes, spermatozoa. Central to this process is the genome‐wide replacement of canonical histones by a unique array of testis‐specific histone variants.^1^, ^2^, ^3^ The incorporation of these histone variants enables the tight regulation of gene expression programs that are critical to spermatogenetic processes such as DNA repair and meiotic recombination.^1^, ^2^, ^4^ These histone variants are replaced by transition proteins, which are then replaced with protamines.^5^, ^6^, ^7^ The tight wrapping of the chromatin around protamines results in a highly condensed structure that fits into the small sperm nucleus. Of the many testis‐specific histone variants that have been identified, the testis‐specific histone H2B variant (TH2B) and the testis‐specific histone H2A variant (TH2A) are of particular interest, because the incorporation of these variants into the nucleosome core particle results in a globally open, more accessible chromatin structure.^8^, ^9^, ^10^, ^11^ The genes encoding for mouse TH2B and TH2A are *Hist1h2ba* and *Hist1h2aa* (Mouse Genome Informatics), respectively. These genes are localized adjacently on chromosome 13 with a shared promoter between them.^12^, ^13^, ^14^ Despite the importance of TH2B and TH2A in regulating chromatin dynamics during spermatogenesis, we are only beginning to understand the role that these variants play during male germ cell development.

Prospermatogonia are the most primitive germ cell population within the mouse testis, and shortly after birth this population of cells transitions into one of two different subpopulations: (1) they can transition directly into differentiating spermatogonia that will contribute to the first cohort of sperm that is produced, otherwise known as the first round of spermatogenesis, or (2) they can transition into the undifferentiated spermatogonial pool, which includes within it the spermatogonial stem cells.^15^, ^16^, ^17^ This second population of cells serves as the progenitors for the subsequent rounds of spermatogenesis. In addition to differences in the progenitor populations from which the first and subsequent rounds of spermatogenesis are derived, numerous studies have also provided evidence of functional and epigenetic differences. Specifically, it has been shown that the cell cycle progression of germ cells within the immature mouse, rat, and hamster testis was accelerated by ~2.5 days compared with that of the adult.^18^, ^19^ Additionally, another study showed that repressive chromatin markers (ie, H3K9me2, H3K9me3, DNMT3A2, and DNMT3B) were absent in spermatogonia with a ZBTB16‐positive, CKIT‐negative (undifferentiated) identity and were present in spermatogonia with a ZBTB16‐negative, CKIT‐positive (differentiating) identity.^20^ Collectively, these findings contribute to the hypothesis that spermatogonia within the neonate are functionally and epigenetically distinct from those within the adult.

Human TH2B and TH2A exhibit high sequence homology with their mouse counterparts, and *TH2B* mRNA and TH2B protein have been localized in the human testis.^21^, ^22^, ^23^ Of interest, mice deficient in *Th2b* showed no obvious spermatogenic defects.^14^ Although no single knockout model of *Th2a* currently exists, the double knockout of *Th2b* and *Th2a* results in sterile mice,^24^ suggesting that TH2B and TH2A function together during spermatogenesis. It is generally accepted that TH2B and TH2A are incorporated into the germ cell chromatin beginning in the spermatocyte stage.^14^, ^25^, ^26^, ^27^ However, there is evidence suggesting that TH2B may be expressed in the undifferentiated spermatogonia. Specifically, Choi and Chae^12^ detected the presence of *Th2b* mRNA in the testes of 6‐day‐old rats, which contain mainly spermatogonia, suggesting that *Th2b* may be expressed in premeiotic germ cells. Another group of researchers was able to detect TH2B protein in spermatogonia within testicular tissue obtained from infertile men, containing only Sertoli cells and spermatogonia.^22^ Although these data are suggestive, it has not been definitely shown if TH2B and/or TH2A are expressed before meiosis in mice.

The goal of the present study was to examine and establish the localization profile of TH2B and TH2A within the neonatal mouse testis. In this report, we also demonstrate that TH2B is differentially regulated in the neonatal compared with the adult testis. Our results highlight the significance of these histone variants in distinguishing the chromatin dynamics of the neonate compared with the adult mouse testis.

## 2 RESULTS

### 2.1 TH2B and TH2A are present in the neonatal mouse testis

**Figure 1:**
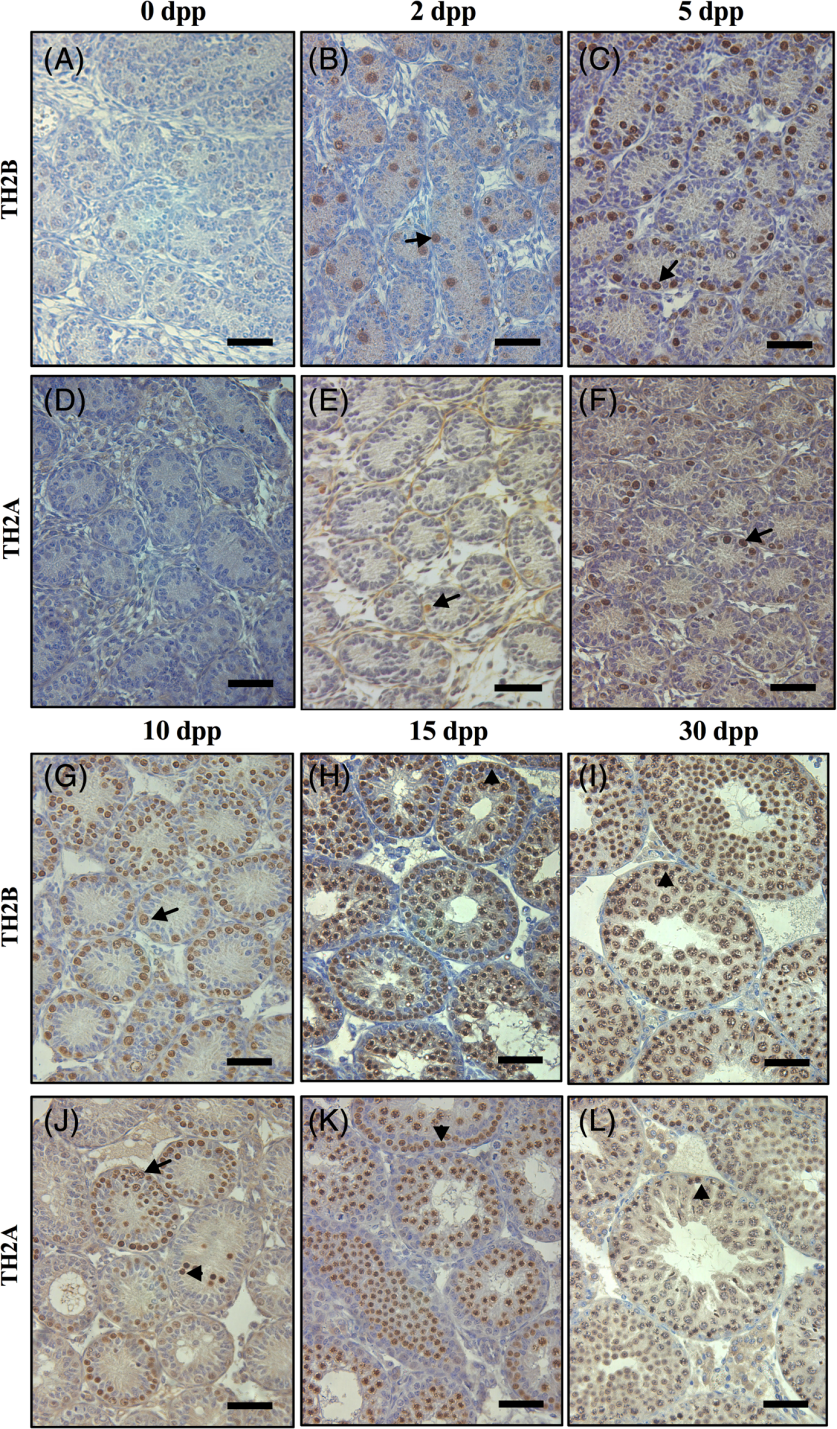
TH2B and TH2A are expressed in the neonatal testis. Representative testis sections are immunostained with antibodies against TH2B and TH2A. Cells immunopositive for TH2B (A‐C,G‐I) and TH2A (D‐F,J‐L) are indicated by brown precipitate. Arrows denote immunopositive spermatogonia, and arrowheads denote immunopositive spermatocytes. Scale bars = 100 μm

To determine the onset of TH2B and TH2A expression in the neonatal mouse testis, we conducted an immunohistochemistry (IHC) study using a developmental testis age series (Figure 1). TH2B immunopositive spermatogonia were first detected in the 2 days postpartum (dpp) testis, and the number of immunopositive spermatogonia steadily increased through 10 dpp (Figure 1B,C,G). By 15 dpp, when the population of primary spermatocytes is fully established in the seminiferous tubules, we were unable to detect TH2B immunopositive spermatogonia. However, the population of primary spermatocytes was immunopositive and this pattern of expression was maintained at 30 dpp (Figure 1H,I). TH2A immunopositive spermatogonia were also detected beginning in the 2 dpp testis and we were able to continue to detect TH2A immunopositive spermatogonia through 10 dpp (Figure 1E,F,J).

**Figure 2:**
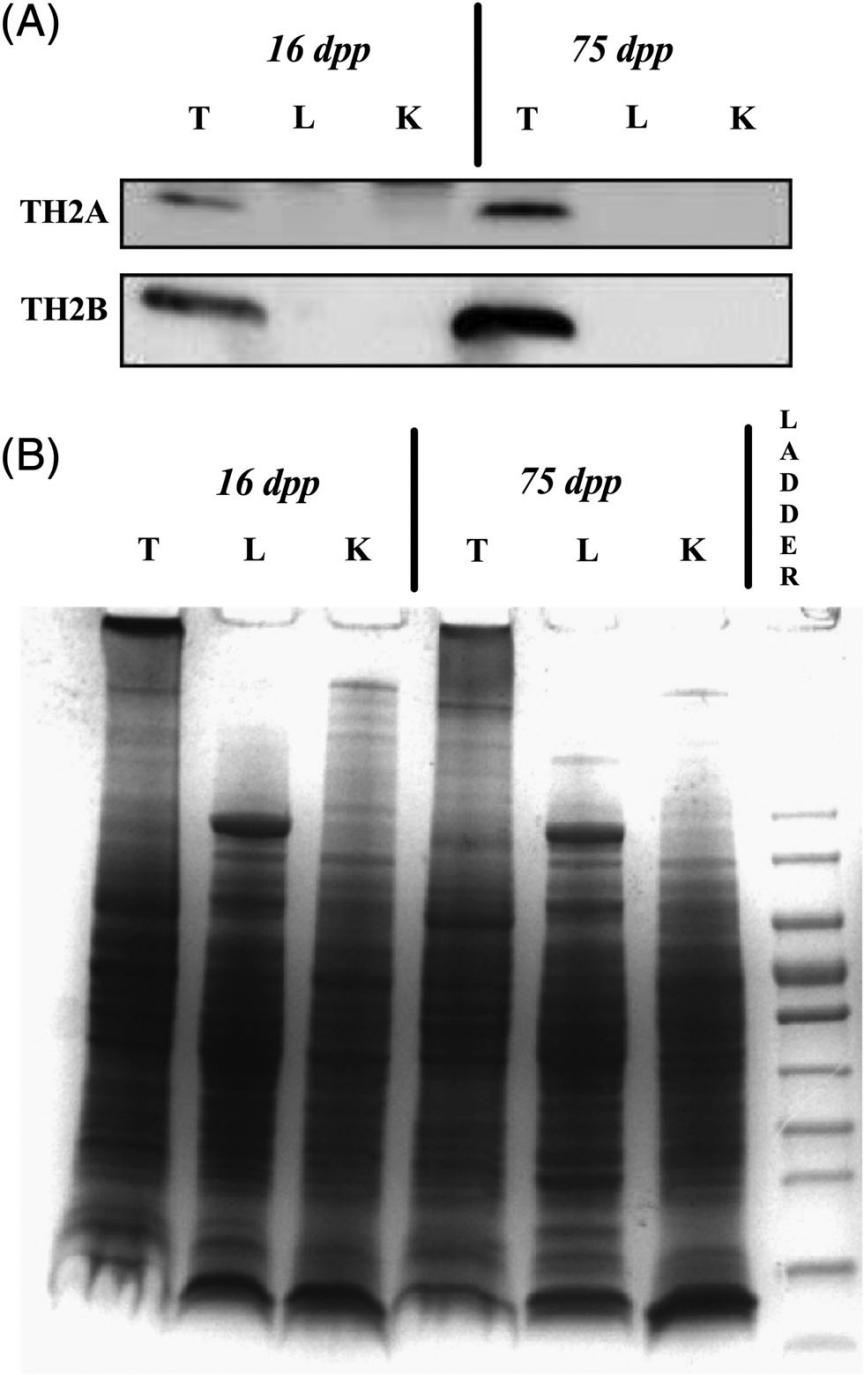
Western blot analysis of TH2A and TH2B. A, Representative Western blots for TH2A and TH2B on testis (T), liver (L), and kidney (K) protein lysates from 16 and 75 dpp mice. The expected size of TH2A and TH2B is 17 kDa. B, Representative Coomassie blue staining used to ensure equal loading of the protein samples

Similar to TH2B, we were unable to detect TH2A immunopositive spermatogonia at 15 and 30 dpp, but TH2A immunopositive spermatocytes were detected at both of those age points (Figure 1K,L). In addition to the verification performed by the manufacturers of the antibodies (Table 1), we also performed Western blots on testis, liver, and kidney protein lysates from 16 and 75 dpp animals (Figure 2A). We were able to detect a band for TH2A and TH2B only in the testis protein lysates at the anticipated weight of 17 kDa (Figure 2A). No signal could be detected in the liver or kidney protein lysates, suggesting that the antibodies detected the testis‐specific variants and not somatic variants (Figure 2A).

**Table 1:** Primary antibodies

Primary antibodies	Manufacturer & identification number	Primary antibody dilution	Notes
Anti‐TH2A	Shinagawa Laboratory, RIKEN Tsukuba Institute	1:500	Shinagawa et al. (2014) demonstrated by Western blot with purified recombinant histone proteins expressed in E. coli that the generated anti‐TH2A antibody detected TH2A but not H2A. In the same research article, the authors also performed a Western blot on adult testis extracts from wild‐type and Th2a^‐/‐^Th2b^‐/‐^ knockout mice and showed no detection of TH2A in the knockout mice.
Rabbit anti‐histone H2B testis‐specific (TH2B)	ABCAM, ab23913	1:1000	The specificity of the anti‐TH2B antibody was tested by Western blot by the manufacturers. A band was detected only when testis samples were used and not when HeLa whole cell lysates were used.
Rabbit Anti‐PLZF (ZBTB16)	Santa Cruz Biotechnology, sc‐22839	1:500	Busada et al. (2014) and Niedenberger et al. (2015) demonstrated by immunohistochemistry that the anti‐PLZF (ZBTB16) antibody marked a population of cells which morphologically resemble undifferentiated spermatogonia.

### 2.2 TH2B and TH2A are present in the undifferentiated spermatogonia

**Figure 3:**
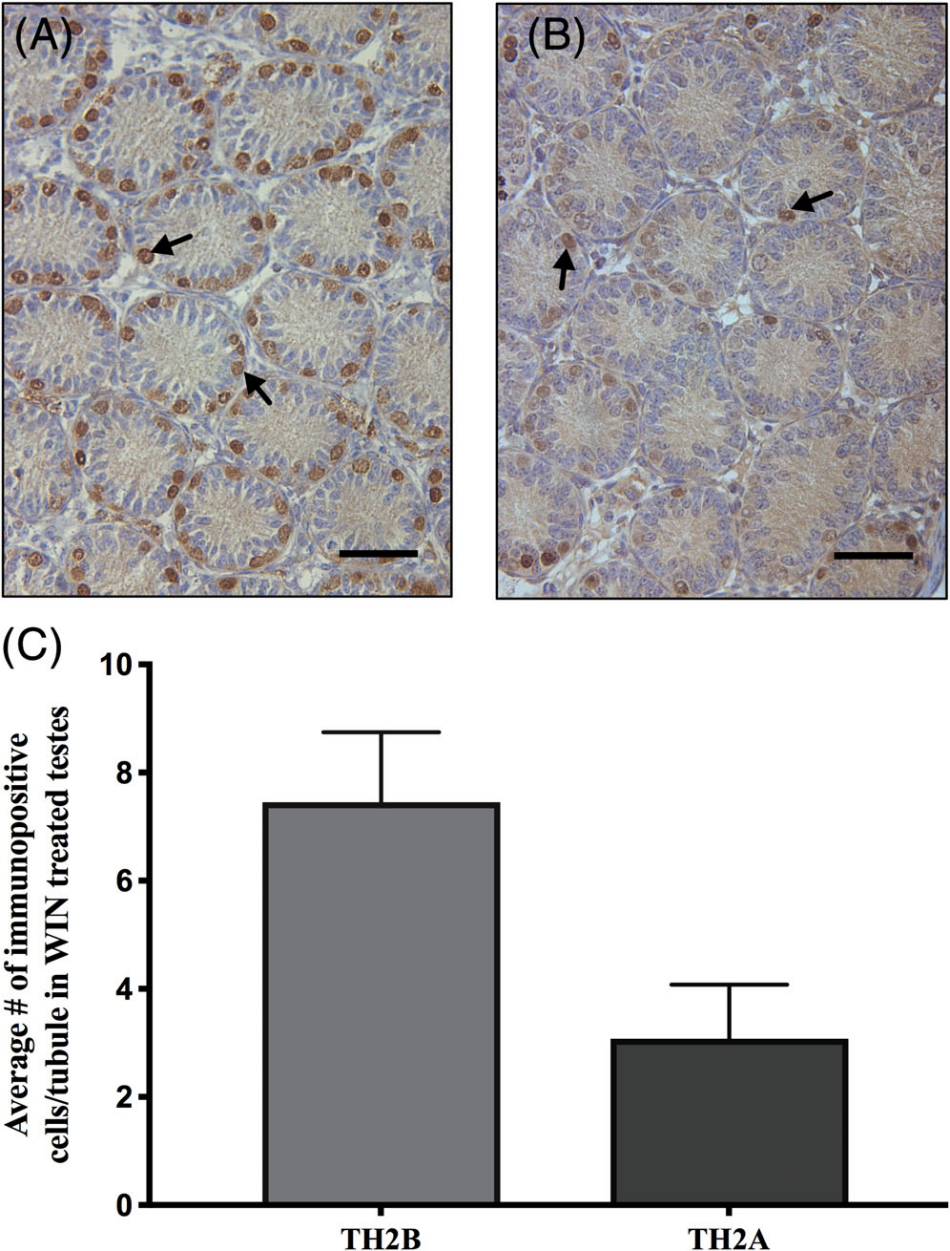
TH2B and TH2B are expressed in WIN treated testes. Representative testis sections from WIN treated mice immunostained for TH2B (A) and TH2A (B). Immunopositive cells are stained in brown (arrows). C, Quantification of the average number of immunopositive cells in WIN treated testes. The average number of immunopositive cells per tubule is shown on the Y‐axis. The histone variant of interest is displayed on the X‐axis. Scale bars = 100 μm

Because previous publications indicated that TH2B and TH2A were expressed beginning in the spermatocyte population,^5^, ^14^, ^27^, ^28^, ^29^ we were surprised to identify premeiotic spermatogonia that were immunopositive for both TH2B and TH2A in the neonatal testis. To investigate if TH2B and TH2A are expressed in the undifferentiated spermatogonial population, we treated animals with the compound WIN 18446 (WIN) to inhibit spermatogonial differentiation and produce a testis‐enriched with undifferentiated spermatogonia.^30^ IHC demonstrated the presence of TH2B and TH2A immunopositive spermatogonia in the WIN treated testes (Figure 3A,B). Quantification revealed that there were approximately 7.4 ± 1.3 and 3.1 ± 1 TH2B and TH2A immunopositive spermatogonia per tubule, respectively (Figure 3C). TH2B expression in the WIN treated testes was approximately 2.4‐fold higher than TH2A (Figure 3C).

### 2.3 TH2B co‐localizes with ZBTB16 in WIN treated testes

**Figure 4:**
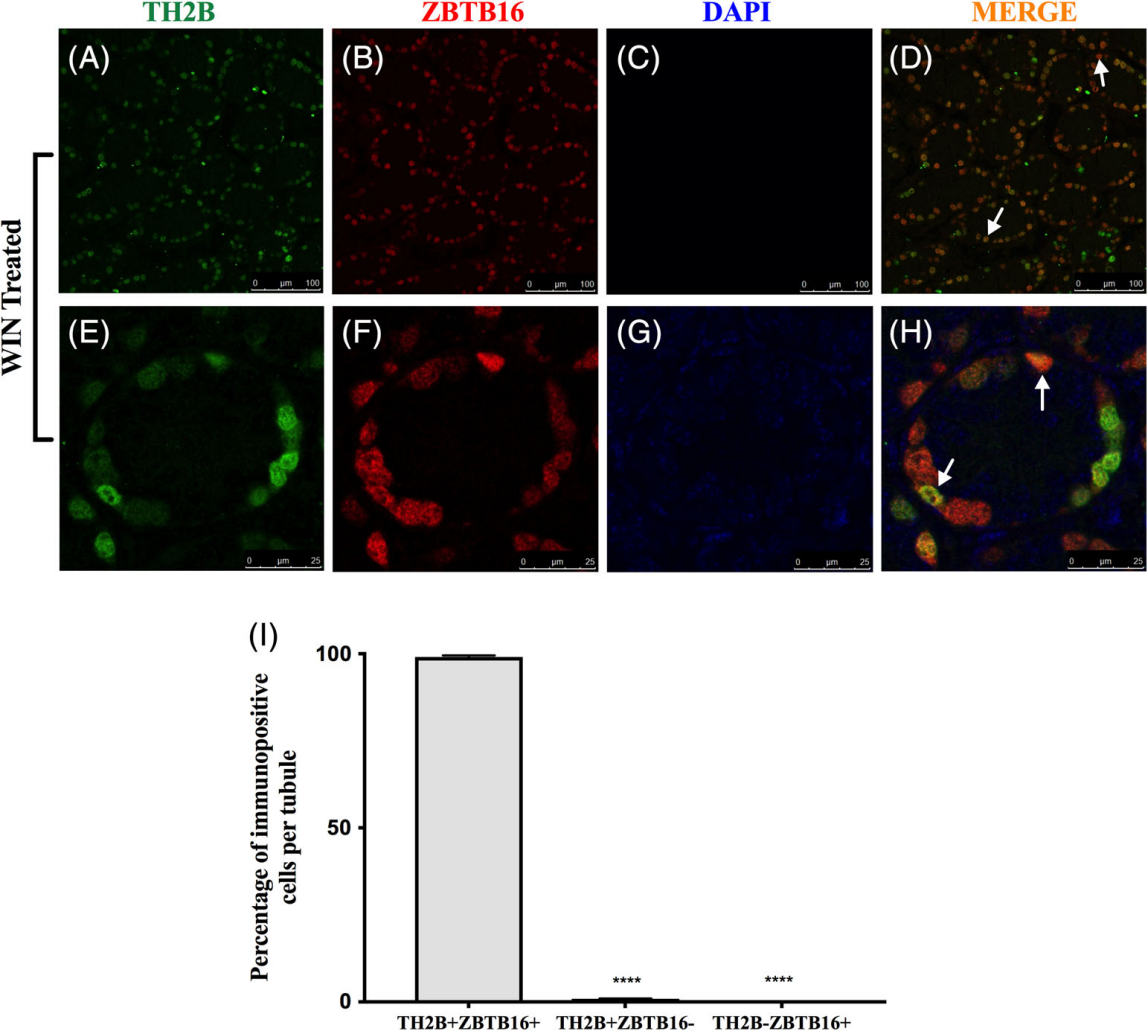
TH2B and ZBTB16 co‐localize in WIN treated testis. Representative co‐immunofluorescence staining of TH2B (A,E), ZBTB16 (B,F), DAPI (C,G), and merged images (D,H) in testis sections from WIN treated mice. White arrows denote TH2B and ZBTB16 co‐localization (D,H). I, Quantification of the percentage of TH2B + ZBTB16+, TH2B + ZBTB16‐, and TH2B‐ZBTB16+ immunopositive cells per tubule. Asterisks represent the statistical difference of a group compared to the TH2B+ ZBTB16+ group. **** *P* < 0.0001

TH2B and TH2A were expressed within WIN treated testes, suggesting that these histone variants may be expressed in the undifferentiated population in the neonate. To clarify the degree to which these histone variants co‐localize with the undifferentiated population in WIN treated testes, we performed an immunofluorescence study to co‐localize TH2B and TH2A with an undifferentiated marker, ZBTB16.^31^, ^32^ Because the primary antibodies used in this study were generated in the rabbit, we used a modified double immunofluorescence protocol to distinguish primary antibodies raised in the same species. Double immunofluorescence staining of TH2B and ZBTB16 demonstrated near perfect co‐localization of the two markers in WIN testes (Figure 4A‐H). Quantification of the number of immunopositive cells revealed that 99.1% of the spermatogonia analyzed were TH2B + ZBTB16+, while 0.9% and 0% of the spermatogonia where either TH2B + ZBTB16‐ or TH2B‐ZBTB16+, respectively (n = 3; Figure 3I). Based on the location and cellular morphology of the small percentage of TH2B + ZBTB16‐ cells in the WIN treated testes, those TH2B + ZBTB16‐ cells were most likely primary spermatocytes that had escaped the synchronization procedure. We attempted to co‐localize TH2A with ZBTB16; however, we were unsuccessful as the TH2A antibody used in this study was not nearly as robust as the commercially available antibodies for TH2B and ZBTB16 and we observed a high degree of background staining. As no other sources of the TH2A antibody were presently available, we forewent any additional co‐localization analysis of TH2A with ZBTB16.

### 2.4 TH2B switches its expression from the ZBTB16+ to the ZBTB16‐ populations

**Figure 5:**
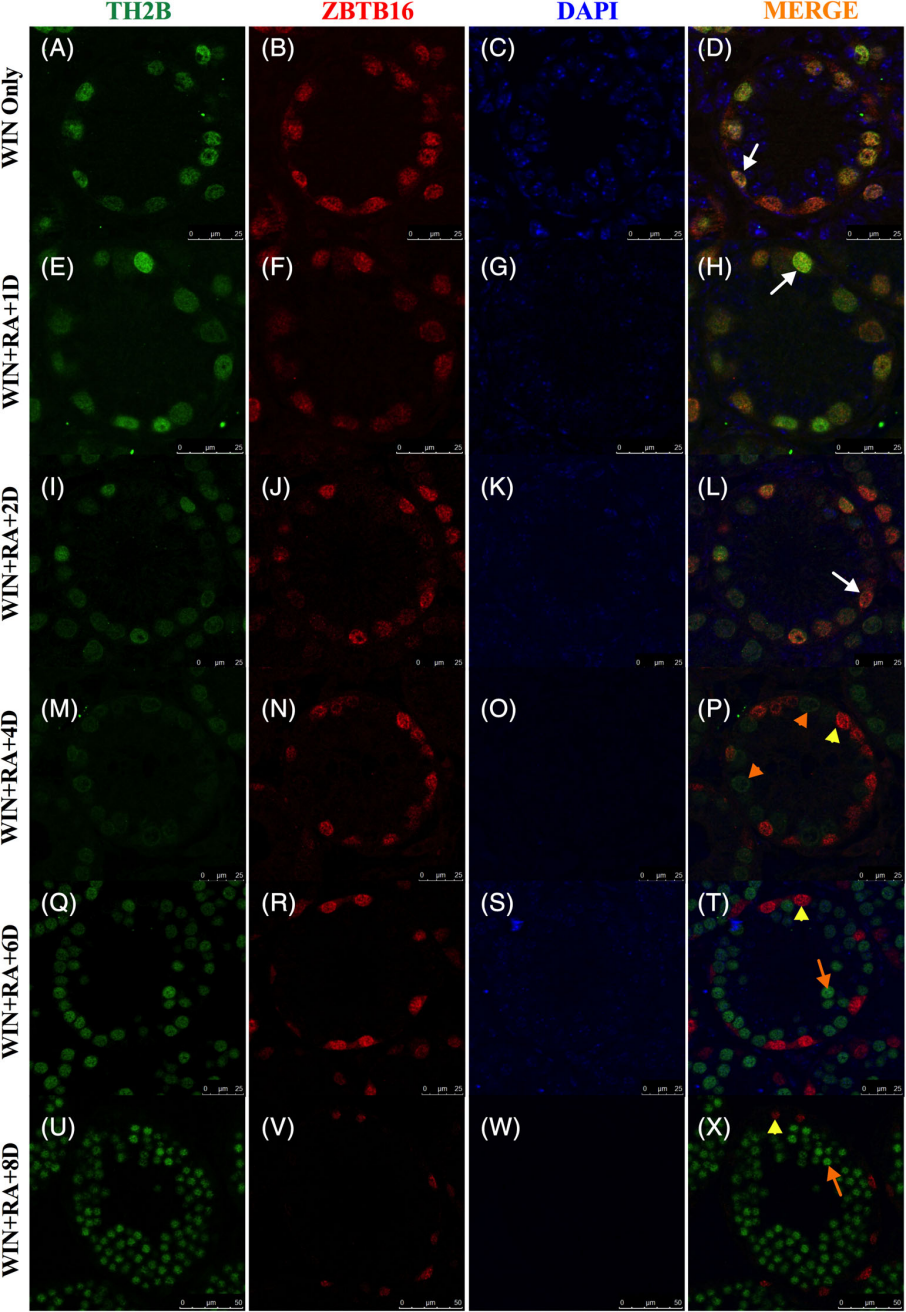
Co‐immunofluorescence staining of TH2B expression in WIN+RA synchronized testes. Representative testis sections immunostained for TH2B (A,E,I,M,Q,U), ZBTB16 (B,F,J,N,R,V), DAPI (C,G,K,O,S,W), and merged images (D,H,L,P,T,X). White arrows denote TH2B + ZBTB16+ co‐localized cells, yellow arrowheads denote TH2B‐ZBTB16+ cells, orange arrowheads denote TH2B + ZBTB16‐ spermatogonia, and orange arrows denote TH2B + ZBTB16‐ spermatocytes

Our previously mentioned morphological analysis of TH2B expression in wild‐type testis samples revealed that as the animals aged, there was a progressive change in the localization of TH2B from the spermatogonia to the spermatocyte population. To obtain a better understanding of when TH2B switched its expression from the undifferentiated population to the primary spermatocytes, we used an established synchronization protocol to first enrich the testis with undifferentiated spermatogonia using WIN, followed by an *all‐trans*‐retinoic acid (*at*RA) injection to trigger the simultaneous differentiation of the undifferentiated population.^30^ Animals were killed at 0 hr (WIN‐only), 1, 2, 4, 6, and 8 days following the *at*RA injection (Figure 5).

**Figure 6:**
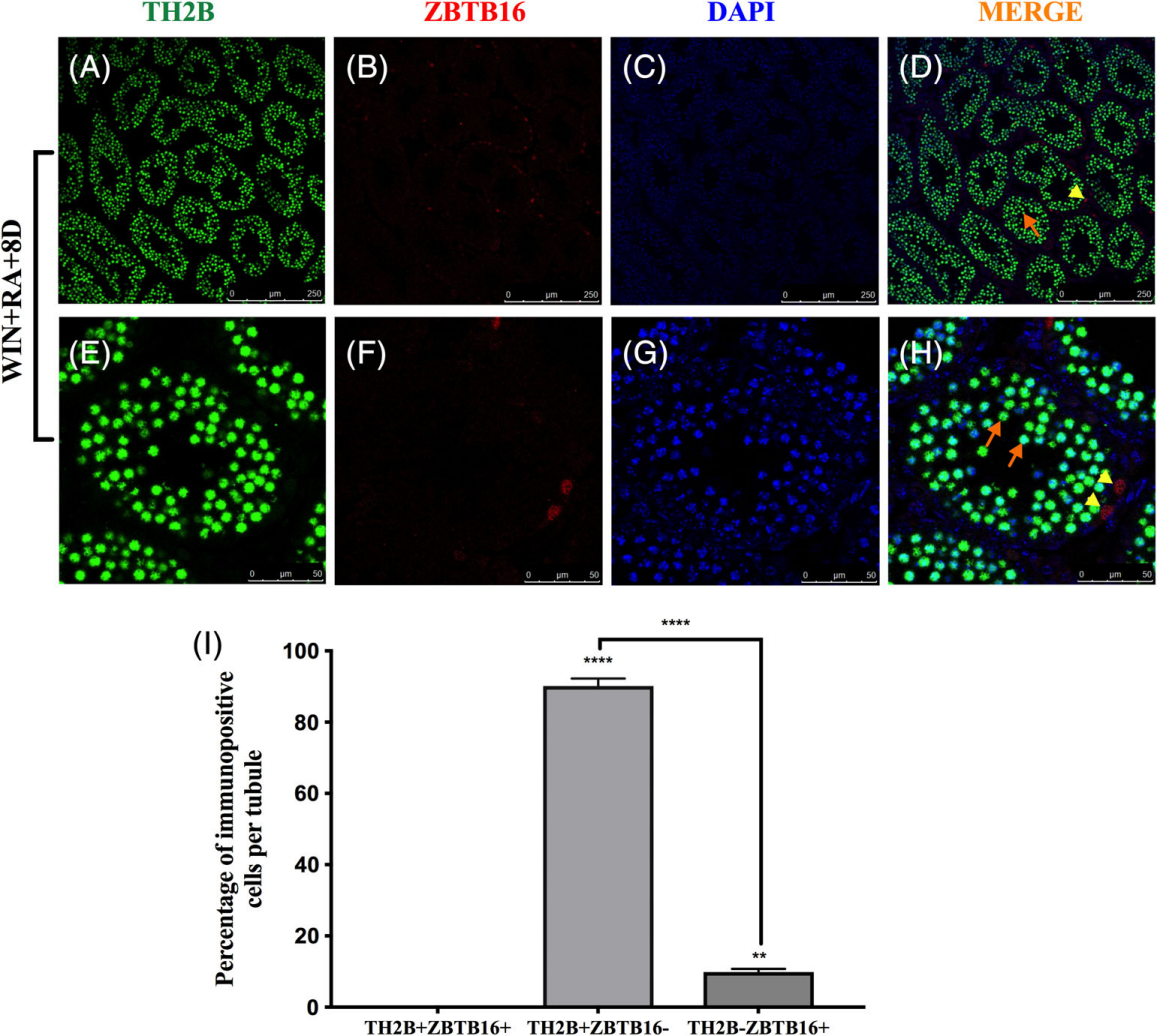
TH2B and ZBTB16 do not co‐localize in WIN+RA + 8D treated mice. Representative co‐immunofluorescence staining of TH2B (A,E), ZBTB16 (B,F), DAPI (C,G), and merged images (D,H). Orange arrows denote TH2B+ ZBTB16‐ spermatocytes, and yellow arrowheads denote TH2B‐ ZBTB16+ spermatogonia. I, Quantification of the percentage of TH2B + ZBTB16+, TH2B + ZBTB16‐, and TH2B‐ZBTB16+ immunopositive cells per tubule. Asterisks represent statistical difference of a group compared to the TH2B+ ZBTB16+ group. Black lines denote the statistical significance between other groups. ** *P* < 0.001 and **** *P* < 0.00001

As expected, there was near perfect co‐localization of TH2B with ZBTB16 in the WIN‐only treated samples (Figure 5A‐D). Co‐localization of TH2B with ZBTB16 steadily declined at 1, 2, and 4 days following the *at*RA injection (Figure 5E‐P). By 6 and 8 days following the *at*RA injection, there was a prominent separation of TH2B and ZBTB16 signals, revealing two distinct populations, a TH2B+ ZBTB16**‐** and a TH2B‐ZBTB16+ population (Figures 5Q‐X and 6A‐H). The timing of this switch in the expression TH2B coincides with the appearance of the preleptotene spermatocytes in the synchronized testis.^33^ At 8 days after WIN treatment and *at*RA injection (WIN+RA + 8), the percentage of cells immunopositive for both TH2B and ZBTB16, or either individually was determined. Zero percent of the spermatogonia were TH2B + ZBTB16+, while 90.1% and 9.9% were TH2B+ ZBTB16**‐** or TH2B**‐**ZBTB16+, respectively (n = 3; Figure 6I).

### 2.5 TH2B is present in both stem and progenitor populations in the neonatal testis

**Figure 7:**
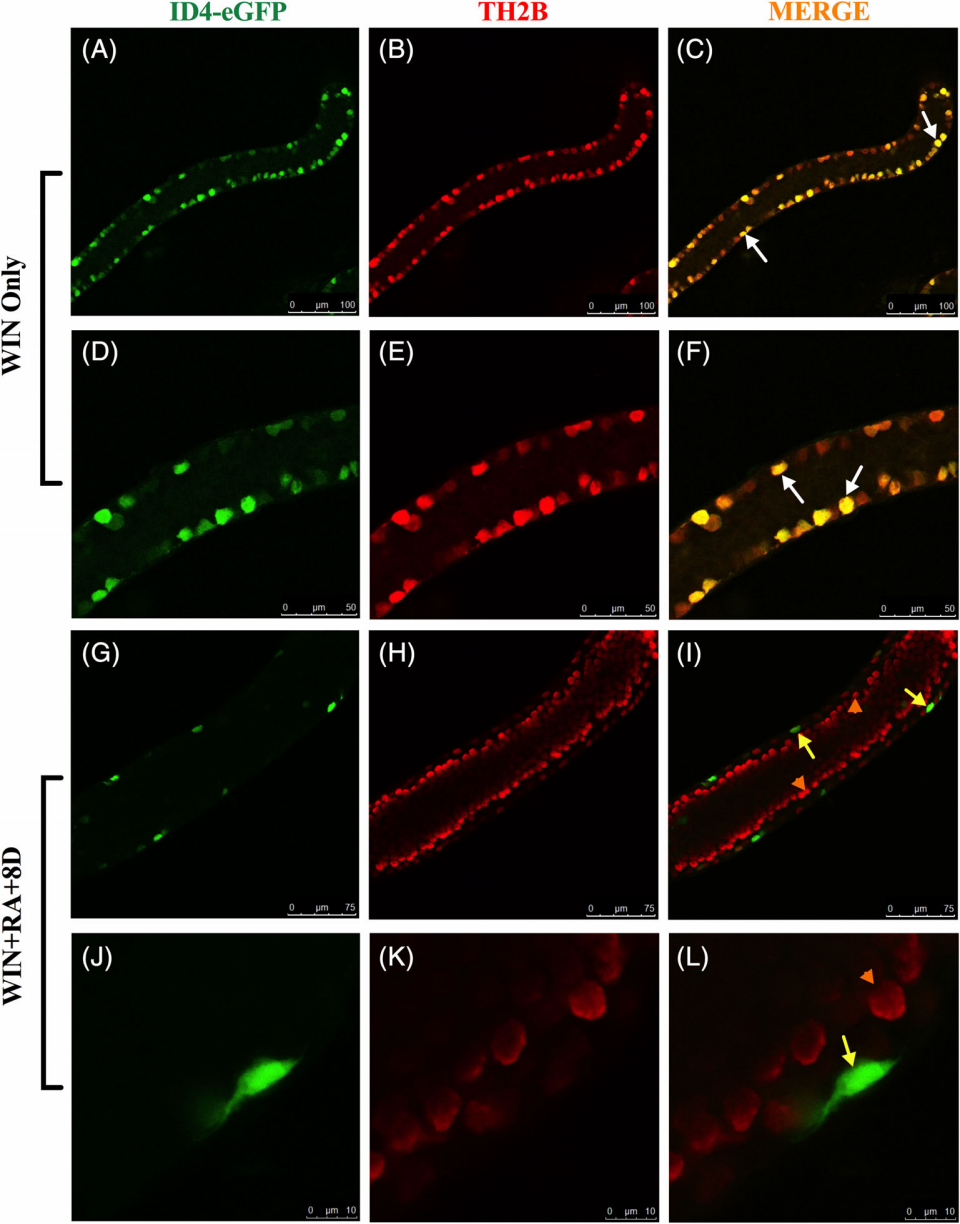
Whole‐mount immunofluorescence staining of TH2B and ID4‐eGFP. Representative tubules from WIN only treated mice stained for ID4‐eGFP (A,D), TH2B (B,E), and merged images (C,F). Tubules from WIN+RA + 8D treated mice stained for ID4‐eGFP (G,J), TH2B (H,K), and merged images (I,L). White arrows denote TH2B + ZBTB16+ cells, orange arrowheads denote TH2B + ZBTB16‐ cells, and yellow arrows denote ID4‐eGFP positive cells

ZBTB16 marks both the stem and progenitor cells that comprise the undifferentiated spermatogonia population.^17^, ^31^, ^32^, ^34^ To analyze whether TH2B was expressed in the spermatogonial stem cell pool, we investigated the expression pattern of TH2B with ID4‐eGFP, a putative stem cell marker.^35^ To achieve this, we performed a whole‐mount immunofluorescent study of ID4‐eGFP and TH2B in WIN‐only and WIN+RA + 8 days treated testes (Figure 7). In the WIN‐only treated testes, ID4‐eGFP expression co‐localized with that of TH2B (Figure 7A‐F). However, in the WIN+RA + 8D treated testes, there was a separation of ID4‐eGFP signals from those of TH2B (Figure 7G‐L).

### 2.6 Switch in TH2B expression occurs in the wild‐type mouse testis

**Figure 8:**
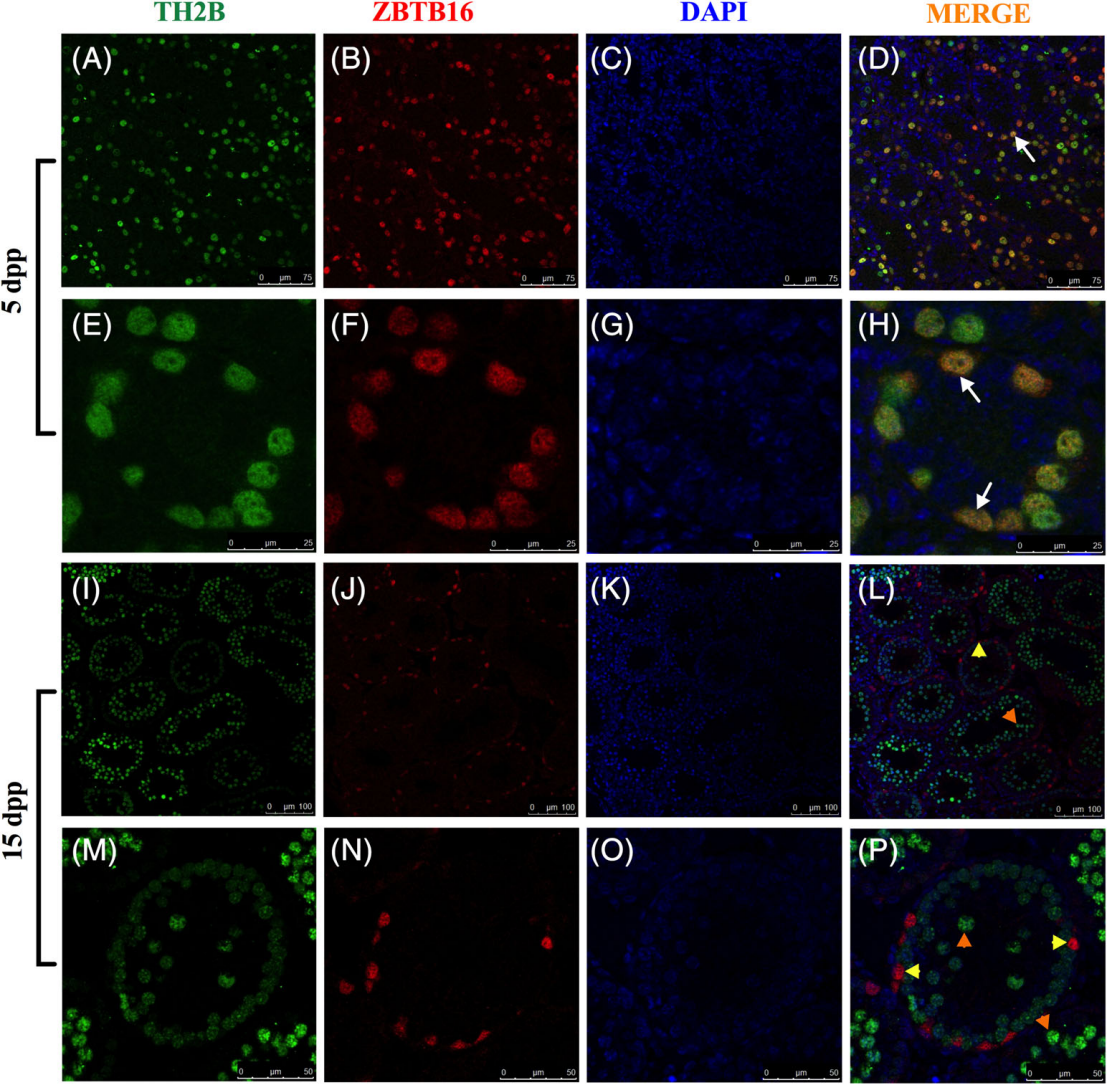
Representative co‐immunofluorescence of TH2B and ZBTB16 in sections from wild‐type mice aged 5 and 15 dpp. Immunofluorescence staining in 5 dpp mice for TH2B (A,E), ZBTB16 (B,F), DAPI (C,G), and merged images (D,H). Immunofluorescence staining in 15 dpp mice for TH2B(I,M), ZBTB16 (J,N), DAPI (K,O), and merged images (L,P). White arrows denote TH2B+ ZBTB16+ spermatogonia, yellow arrowheads denote TH2B‐ZBTB16+ spermatogonia, and orange arrowheads denote TH2B + ZBTB16‐ spermatogonia

Double immunostaining for TH2B and ZBTB16 in 5 dpp wild‐type mouse testis revealed a predominant ZBTB16 + TH2B+ population (Figure 8A‐H). However, transitional cells that were TH2B+ and faintly ZBTB16+ or ZBTB16+ and faintly TH2B+ were also observed. When the population of primary spermatocytes is fully established at 15 dpp, immunostaining for TH2B and ZBTB16 revealed two distinct populations, a ZBTB16 + TH2B**‐** and a ZBTB16**‐**TH2B+ population (Figure 8I‐P).

## 3 DISCUSSION

In the present study, we examined the localization pattern of the testis‐specific histone H2B variant TH2B and the testis‐specific histone H2A variant TH2A within the neonatal mouse testis. We found that TH2B and TH2A are expressed in the undifferentiated population. Importantly, we also showed that TH2B was expressed in stem cells and progenitor spermatogonia (ID4‐eGFP^+^ and ZBTB16^+^) within the neonatal testis. However, when the first spermatocytes appear, TH2B was no longer detected in the ID4‐eGFP+ and ZBTB16+ population and was instead detected within spermatocytes. These results contribute to the growing body of evidence demonstrating epigenetic and functional differences between the neonatal and adult mouse testis.

In our initial experiments, we were interested in determining the onset of TH2B and TH2A expression in the neonatal mouse testis through the use of a testis age series. Our IHC analysis revealed that TH2B and TH2A could be detected as early as 2 dpp in spermatogonia. This is contrary to previous reports demonstrating that TH2B and TH2A are up‐regulated beginning in the 8 dpp testis in the spermatocyte population.^14^ These differences likely arise from variation in fixation protocols, immunostaining procedures as well as mouse strains used. However, it is worth noting that a majority of the analyses demonstrating that TH2B is localized initially to spermatocytes was discovered using the adult rat.^5^, ^14^, ^27^, ^28^, ^29^ These data are consistent with our findings that after the first spermatocytes appear, TH2B switches its localization from the spermatogonia to the spermatocyte population. Our findings demonstrate that in addition to the published expression of TH2B in adult spermatocytes, TH2B is also expressed in spermatogonia during the first round of spermatogenesis.

Additional studies to investigate the localization pattern of TH2A with ZBTB16 were attempted in our laboratory. However, we were unsuccessful in our attempt to detect TH2A expression using our modified double‐immunofluorescence protocol, as the primary antibody for TH2A was not nearly as robust as the commercially available antibodies for TH2B and ZBTB16. However, our finding of TH2A immunopositive cells in spermatogonia within the WIN treated testis, and within the spermatocyte population from IHC analysis of testis sections from older mice, suggests that TH2A may also display a switch in its expression in a manner similar to TH2B. Future studies on the expression profile TH2A may elucidate an additional role for this variant in the undifferentiated spermatogonial population.

Historically stem and progenitor populations in the neonatal testis are described as having an open chromatin state devoid of heterochromatin.^36^ This open chromatin state may be made possible by the incorporation of histones and modifications which loosen/open the chromatin structure. Therefore, the normal function of TH2B, and potentially of TH2A, may be to globally mediate nucleosome instability. This role for TH2B and TH2A is also supported by biochemical studies demonstrating that nucleosomes containing TH2B and TH2A are more sensitive to DNase and MNase digestions, due to a reduction in the number of strong hydrogen bonds resulting from more loosened, open chromatin.^37^, ^38^ Although the biological significance of this loosened/open chromatin within the neonatal testis is presently unclear, this open chromatin state could contribute to the multipotency of the germ cell lineage. In the neonatal testis, this globally open chromatin structure could allow for the germ cell genome to be in a transcriptionally active state, which would enable the timely activation of essential genes required for the initial differentiation of prospermatogonia into multiple lineages.

One of the major questions with regard to the regulation of TH2B is how this differential expression between the first and subsequent rounds is established. Choi and Chae^12^ showed that CpG sites in the promoter regions of the TH2B gene were methylated in somatic cells and hypomethylated in germ cells. This epigenetic mechanism of regulation is what establishes the differential gene expression of TH2B in germ cells compared with somatic cells. However, studies have also shown that this germ cell‐specific pattern of hypomethylation is present at all stages of spermatogenesis.^12^, ^28^, ^39^ This hypomethylated state of the gene may explain why we observed TH2B expression in the neonate. It is tempting to hypothesize that an additional epigenetic mechanism (ie, DNA methylation or histone posttranslational modifications) may be the reason for the differential expression of TH2B in the first compared with subsequent rounds of spermatogenesis. Further studies aimed at uncoupling the relationship between these epigenetic mechanisms and histone expression, particularly of TH2B, may elucidate how the expression of this protein is differentially regulated at different stages of spermatogenesis.

Collectively, our results support previous conclusions that spermatogonia in the neonatal testis are inherently distinct from spermatogonia in the adult. We have shown that two histone variants, TH2B and TH2A, are expressed in undifferentiated spermatogonia within the neonatal testis. Our findings also demonstrate that TH2B is differentially expressed in the first round compared with subsequent rounds of spermatogenesis. Further studies will need to be performed to determine the degree to which TH2A follows the same trends as TH2B. However, we believe that these basic findings help to better understand the roles that epigenetic factors, such as histone variants, play in the regulation of mammalian spermatogenesis.

## 4 EXPERIMENTAL PROCEDURES

### 4.1 Animals and tissue processing

All animal experiments were approved by the Washington State University Animal Care and Use Committees and were performed in accordance with the National Institutes of Health Guide for the Care and Use of Laboratory Animals. Breeding colonies of the C57BL/6‐129 mouse line and the ID4‐eGFP (LT‐11B6) transgenic reporter mouse line^35^ were used in the following studies. All mouse lines were maintained in a temperature‐ and humidity‐controlled facility with food and water provided ad libitum.

Testes from three to five C57BL/6‐129 animals per age were used for immunohistochemical and immunofluorescence analysis. Depending on the age of the animal, testes were fixed in Bouins fixative (M7831‐88, EMD Millipore) for 30 min to 2 hr at room temperature. The tissue was then dehydrated in a graded ethanol series and embedded in paraffin wax. Sections of 4‐5 μm with at least 20 μm between each cross‐section were placed on Superfrost Plus microscope slides (12‐5550‐15, Fisher Scientific).

Testes from ID4‐eGFP mice were used in the whole‐mount immunofluorescence studies.^35^ Testis tissue was detunicated, gently dissociated and fixed in 4% paraformaldehyde (P6148, Sigma‐Aldrich) for 2 hr at 4°C. Following fixation, the tissue was washed and stored in 1× phosphate buffered saline (PBS) until staining was performed. Testes from three to four ID4‐eGFP animals were analyzed per treatment group.

### 4.2 WIN 18446 and all‐trans‐retinoic acid treatments

Neonatal mice (2 dpp) were pipette fed 100 mg/kg body weight WIN 18446 suspended in 1% gum tragacanth for 7 consecutive days.^30^ At 9 dpp (day 8 of treatment), testes from some of the treated animals were collected as the time zero samples (WIN‐only) and the rest were administered an intraperitoneal (IP) injection of *all‐trans*‐retinoic acid (R2625, Sigma‐Aldrich) at a dose of 200 mg/kg suspended in 10 μL dimethyl sulfoxide.^30^ *All‐trans*‐retinoic acid injected animals were left to recover for various intervals between 4 hr to 8 days following the *at*RA injection, at which point the testes were removed and processed as described for IHC, immunofluorescence, or whole‐mount analyses.

### 4.3 IHC

IHC was performed as previously described with minor modifications.^40^ Briefly, testis sections underwent a rehydration consisting of two xylene washes (5 min each), a graded ethanol series (100%, 100%, 90%, and 70% for 5 min each), and antigen retrieval in 0.01 M citrate buffer (NaCitrate dihydrate diluted in ddH_2_O, pH 6, and > 90°C in a microwave for 7.5 min). To quench endogenous peroxidases, tissue sections were incubated in 3% hydrogen peroxide (H_2_O_2_) for 5 min. To minimize nonspecific binding of secondary antibodies, preincubation was performed for 30 min with 5% normal goat serum (NGS) suspended in phosphate buffer (PB) (5% NGS/PB). PB consisted of 0.1% bovine serum albumin, and 1× PBS (137 mM NaCl, 2.7 mM KCL, 10.1 mM Na_2_HPO_4_, and 1.8 mM KH_2_PO_4_). The primary antibodies used in these experiments are listed in Table 1. Primary antibodies were diluted in 5% NGS/PB and incubated on tissue sections in humidified chambers at room temperature overnight.

Control sections were incubated without the primary antibody. Three 1× PBS washes (5 min each) were performed between all subsequent incubations. The incubations were also performed in a humidified chamber at room temperature for 1 hr. Primary antibody binding was detected using the ready‐to‐use biotinylated goat anti‐rabbit antibody included in the Histostain‐Bulk SP IHC Kit (959943B, ThermoFisher). To enable visualization of the secondary antibodies, sections were incubated with a streptavidin‐conjugated horseradish peroxidase (HRP) enzyme also provided within the Histostain‐Bulk SP IHC Kit (959943B, ThermoFisher). Antibody binding was visualized using 3,3‐diaminobenzidine (DAB) HRP substrate (34 002, ThermoFisher). The intensity of the DAB staining (detected as a brown precipitate) was monitored through a microscope. All slides were developed for the same amount of time (~40 sec) and the reaction was quenched in water. Nuclei were counterstained using Harris hematoxylin stain (HHS128, Sigma‐Aldrich) for 20 sec, followed by dehydration and mounting under glass coverslips in DPX (100503‐834, VWR International).

### 4.4 Co‐immunofluorescence using two rabbit polyclonal immunoglobulin G antibodies

For co‐immunofluorescence studies, testis sections underwent rehydration, antigen retrieval, and an initial blocking in 5% NGS/PB as described above with the exception that the initial xylene washes were 20 min each. Unless otherwise indicated, three 1× PBS washes were performed between all subsequent incubations. All incubations were also performed at room temperature in a humidified chamber for 1 hr. The first rabbit polyclonal antibody (TH2B) was diluted in 5% NGS/PB and incubated overnight. Tissues were then incubated with a biotinylated secondary goat anti‐rabbit (959943B, ThermoFisher), followed by an incubation in AlexaFluor 568 streptavidin conjugate (S11223, ThermoFisher), both diluted in 5% NGS/PB. Tissues were then incubated with 1% normal rabbit serum suspended in PB to saturate open binding sites on the first secondary antibody with rabbit immunoglobulin G (IgG). This step ensured no binding by the second primary antibody (ZBTB16).

To cover the rabbit IgG so that the second secondary antibody would not bind to it, a 3‐hr incubation with unconjugated Fab antibody goat anti‐rabbit (H + L) (40 μL/mL; 811‐1602, Rockland) in PB was performed. Subsequently, the tissues were blocked in 5% normal donkey serum suspended in PB (5% NDS/PB). The second rabbit polyclonal antibody (ZBTB16) was diluted in 5% NDS/PB and incubated overnight. The following day, sections were incubated in AlexaFluor 488 donkey anti‐rabbit (A21206, Life Technologies) diluted in 5% NDS/PB. Slides were then mounted with fluoroshield mounting medium containing 4′,6‐diamidine‐2‐phenylidole‐dihydrochloride (DAPI; ab104139, Abcam). To ensure non‐cross‐reactivity after consecutive staining with two rabbit primary antibodies, the omission of all combinations of antigens (eg, the first primary, second primary or either of the secondary antibodies) was evaluated and compared with the results of the double labeling. A Leica TCS SP5II confocal microscope and the LAS AF software were used to capture all images.

### 4.5 Cell quantification

The spermatogonia and spermatocyte populations were distinguished by (1) the shape and size of the cell nuclei and (2) the location of the cell within the testis tubule. Cells were also identified to be undifferentiated if they were ZBTB16‐, or ID4‐eGFP‐positive immunostaining. All cell quantifications were performed with a minimum of three animals per experiment and a minimum of 200 round testis tubules per animal.

### 4.6 Western blotting

Tissue samples were homogenized on ice to prepare protein samples in RIPA buffer (1% Nonidet P‐40, 0.5% Tween 20, 0.1% sodium dodecyl sulfate [SDS] in 50 mM TrisCl pH 8, 150 mM NaCl, 1 mM ethylenediaminetetraacedic acid, 1 mM TCEP) with protease inhibitors (Protease Inhibitor Cocktail, Sigma P8340). Protein concentration was measured using the BCA protein assay kit (Pierce). Fifty micrograms of each protein sample were loaded onto each lane of an SDS‐polyacrylamide gel electrophoresis gel (BioRad #456‐10 084) with protein size standards (PageRuler Prestained Protein ladder, Thermo Scientific #26616). The gel was run at 100 V for 65 min and then stained with Coomassie blue or transferred to a polyvinyl difluoride membrane at 1.3 A, 25 V for 7 min using a BioRad Trans‐Blot Turbo Transfer system. Membranes were blocked in 5% nonfat dry milk containing TBS for 1 hr. Primary antibodies were diluted in 5% nonfat dry milk, TBS, and 0.1% Tween 20.

Incubation with primary antibodies for TH2B (1 μg/mL) and TH2A (1:1000) was performed at 4°C overnight. Incubation with HRP‐coupled secondary antibodies (goat anti‐rabbit) (1:2500) was performed at room temperature for 2 hr. Antibody binding was detected using HyGLO Quick Spray chemiluminescence HRP antibody detection reagent (Denville Scientific). Membranes were digitally photographed using a Fiji LAS 4000 imager. Each antibody was tested on at least two distinct homogenates for each sample age, with consistent results. For juvenile testis samples, lysates were prepared from testes collected from multiple mice that were pooled. Coomassie blue staining was used to ensure equal loading of protein samples.

### 4.7 Whole‐mount immunofluorescence

Unless otherwise noted, all of the following washes were performed under gentle rotation at 4°C. Seminiferous tubules stored in 1× PBS for whole‐mount immunofluorescence were washed in 0.2% Nonidet P40 Substitute (74 385, Sigma‐Aldrich)/1× PBS for 20 min. A graded methanol series (25%, 50%, 75%, 100%) in PBS‐T (PBS containing 0.1% Tween 20 (P1379, Sigma‐Aldrich) for 5 min each was performed to dehydrate the tubules. This was followed by two washes in PBS‐T for 5 min each for rehydration. Nonspecific binding of the primary antibodies was inhibited by immersing the testis tubules in blocking solution (1% bovine serum albumin/10% donkey serum/PBS‐T) for 1 hr. The tubules were then incubated with the TH2B primary antibody diluted in blocking solution overnight at 4°C with gentle rotation. The tubules were washed three times in PBS‐T for 5 min each.

For detection of ID4‐eGFP, tubules were incubated with an anti‐green fluorescent protein conjugated to fluorescein isothiocyanate (Abcam, ab6662), while detection of TH2B was visualized by incubating the tubules simultaneously in AlexaFluor 568 donkey anti‐rabbit (A10042, ThermoFisher Scientific) diluted in PB for 2 hr with gentle rotation at room temperature. The tubules were washed three times in PBS‐T for 15 min each at room temperature. Tubules were mounted in fluoroshield mounting medium with DAPI (ab104139, Abcam) under glass coverslips. A confocal microscope (Leica TCS SP5II) was used to image whole seminiferous tubules.

### 4.8 Statistics

Data are presented as mean ± SE of the mean. Statistical analysis was determined using a Student's *t*‐test or analysis of variance with a Tukey's post hoc test (Prism; version 7.0c; GraphPad). A *P*‐value of 0.05 or less was considered statistically significant.
